# An Evidence-Based Health Care Knowledge Integration System: Assessment Protocol

**DOI:** 10.2196/11754

**Published:** 2019-03-11

**Authors:** Véronique Nabelsi, Sylvain Croteau

**Affiliations:** 1 Département des sciences administratives Université du Québec en Outaouais Gatineau, QC Canada; 2 Hôpital de Gatineau, Centre intégré de santé et des services sociaux de l’Outaouais Gatineau, QC Canada

**Keywords:** knowledge translation, practice guideline, community medicine, group practice, evidence-based medicine, clinical decision making, educational technology, decision support systems, clinical

## Abstract

**Background:**

The rapid advancements in health care can make it difficult for general physicians and specialists alike to keep their knowledge up to date. In medicine today, there are deficiencies in the application of knowledge translation (KT) in clinical practice. Some medical procedures are not required, and therefore, no value is added to the patient’s care. These unnecessary procedures increase pressures on the health care system’s resources, reduce the quality of care, and expose the patients to stress and to other potential risks. KT tools and better access to medical recommendations can lead to improvements in physicians’ decision-making processes depending on the patient’s specific clinical situation. These tools can provide the physicians with the available options and promote an efficient professional practice. Software for the Evolution of Knowledge in MEDicine (SEKMED) is a technological solution providing access to high-quality evidence, based on just-in-time principles, in the application of medical recommendations for clinical decision-making processes recognized by community members, accreditation bodies, the recommendations from medical specialty societies made available through campaigns such as Choosing Wisely, and different standards or accreditive bodies.

**Objective:**

The main objective of this protocol is to assess the usefulness of the SEKMED platform used within a real working clinical practice, specifically the Centre intégré de santé et des services sociaux de l’Outaouais in Quebec, Canada. To achieve our main objective, 20 emergency physicians from the Hull and Gatineau Hospitals participate in the project as well as 20 patient care unit physicians from the Hull Hospital. In addition, 10 external students or residents studying family medicine from McGill University will also participate in our study.

**Methods:**

The project is divided into 4 phases: (1) orientation; (2) data synthesis; (3) develop and validate the recommendations; and (4) implement, monitor, and update the recommendations. These phases will enable us to meet our 6 specific research objectives that aim to measure the integration of recommendations in clinical practices, the before and after improvements in practices, the value attributed by physicians to recommendations, the user’s platform experience, the educational benefits according to medical students, and the organizational benefits according to stakeholders. The knowledge gained during each phase will be applied on an iterative and continuous basis to all other phases over a period of 2 years.

**Results:**

This project was funded in April 2018 by the Fonds de soutien à l’innovation en santé et en services sociaux for 24 months. Ethics approval has been attained, the study began in June 2018, the data collection will be complete at the end of December 2019, and the data analysis will start in winter 2020. Both major city hospitals in the Outaouais region, Quebec, Canada, have agreed to participate in the project.

**Conclusions:**

If results show preliminary efficacy and usability of the system, a large-scale implementation will be conducted.

**International Registered Report Identifier (IRRID):**

PRR1-10.2196/11754

## Introduction

### Background

The rapid advancements in health care can make it difficult for general physicians and specialists alike to keep their knowledge up to date. In medicine today, there are deficiencies in the application of knowledge translation (KT) in clinical practice. The Canadian Institutes of Health Research (CIHR) defines knowledge translation as “a dynamic and iterative process that includes synthesis, dissemination, exchange and ethically sound application of knowledge to improve the health of Canadians, provide more effective health services and products, and strengthen the health care system” [1].

KT is of critical importance, considering the numerous gaps between what we know and the actual care delivered. The CIHR has come to the conclusion that “it has become clear that the creation of new knowledge often does not, on its own, lead to widespread implementation or impacts on health,” [2].

In fact, the best evidence and best practices promoted by scholarly institutions and organizations are not always implemented, and some patients do not receive the most appropriate treatment. According to the Choosing Wisely campaign, it is estimated that up to 30% of examinations, treatments, and interventions performed in Canada are potentially useless or harmful [3-5]. Some studies demonstrated that 30% of patients in Ontario underwent unnecessary cardiac testing and blood analysis before a low-risk noncardiac surgical intervention [4,6]. Still, other studies have shown that up to 50% of trauma patients passing through the emergency do not receive all the prescribed treatments because of a lack of KT [7]. In other instances, from the expectations in regard, a prescribed test or a specific treatment is not aligned with evidence [8].

### Adoption of Clinical Recommendations and Guidelines Into Practice

Throughout the world, including Canada, it is increasingly recognized that some medical procedures are unnecessary and provide no added value to the treatment [3]. These unnecessary medical procedures, which may not even address patient need, then increases pressures on the health care system’s resources, reduces the quality of care, adds to patient stress, and exposes them to other potential risks [9,10]. The context described leads to the delivery of less efficient care for the patients exposing them to potential risks and stress [11]. Knowledge creation (ie, primary research), knowledge distillation (ie, the creation of systematic reviews and guidelines), and knowledge dissemination (ie, appearances in journals and presentations) are not enough on their own to ensure the use of knowledge in decision making [12]. It might be possible to guide the clinicians with a platform that proposes better KT and knowledge-to-action tools and really permits adoption in the clinical setting of recognized and approved clinical recommendations and guidelines. This has the potential to improve not only patient care but also the doctor-patient relationship which is based on communication, trust, and information exchange.

### Selection and Adaptation of Recommendations

The Choosing Wisely campaign aims to help clinical practitioners and patients engage in dialogue about unnecessary examinations, treatments, and interventions and to make stronger and more efficient choices regarding quality care. This initiative is now implemented in 18 countries across the world, including Canada, where several recommendations have been issued pertaining to a wide range of clinical specialties, which enable an improved information exchange on best practices [13-15]. Several studies have demonstrated the relevance of their implementation in various clinical practices [16-17]. Like the Choosing Wisely campaign, the Institut national d’excellence en santé et en services sociaux (INESSS) mission is to promote clinical excellence and the efficient use of resources in the health and social services sector [18]. KT is also an integral part of their mission in “fostering the implementation of the recommendations and practice guides, using various information, knowledge transfer and awareness tools, p. 3” [19]. The INESSS also develops clinical recommendations and clinical practical guidelines intended for various communities of practice (CoP), supported by evidence, experiential, and contextual data provided by medical professionals [20].

### Problem

The challenge for the health care system is to help physicians adequately implement exemplary practices. For professional guidelines to be implemented and to improve the quality of care, it is essential that doctors become aware of the existence of these practices through appropriate methods of dissemination and implementation in day-to-day practice.

Furthermore, the transfer of theoretical knowledge and practical experience from one context to another is a focus in medical education [21-23]. This is a major concern in professional sectors and is central to the debate surrounding reforms. According to Kontoghiorghes [24], only 10% to 15% of learning translates from training to clinical practice. It is, therefore, essential to focus on the capacity to mobilize and combine knowledge to efficiently address new situations [25]. The mobilization resulting from the creation of a community of practice could promote learning and promote working with other health care professionals to solve concrete clinical problems [25]. Collaborative learning reinforces learned knowledge and encourages practical changes. For this reason, knowledge and expertise sharing between professionals and between clinical CoP is a recognized strategy to bring change to clinical practice [26,27].

Some models proposed in recent years promote the development of CoP and the continuity of patient-centered care [28-33]. These models aim at creating proactive, interdisciplinary professional teams and CoP who interact at various levels of the health care system. Studies have demonstrated the added value through the quality of interventions [34-36] of this organizational system. Integrated CoP would support clinical practitioners across various clinical settings in carrying out their daily activities and developing better patient-centric practices.

A CoP is defined as a group of individuals who are interacting to share information, experiences, models, views, advices, and best practices, as well as to solve problems and extend their knowledge in an area of practice in which they share a common interest [37]. Each member of a CoP is supported by a peer group belonging to an area of expertise or a professional practice where he can ask questions, share, and create new knowledge. Relationships with other CoP can also be established. In that respect, the creation of integrated CoP would enable the support of clinical practitioners in their daily activities as well as the growth and development of best practices. However, to make that a reality, an integrative model favoring the implementation of a knowledge-sharing structure between members of a CoP must be used to promote the emergence of collective intelligence.

Information technology (IT)–enabled CoP can support the knowledge learning and sharing activities within interdisciplinary health care teams. A study based on the Hoyman model demonstrated how computer applications can ensure the continuity and flow of the care [38].

KT tools and access to the guidelines can guide and improve physician decision-making processes depending on specific clinical situations of the patient. These tools can provide them with the available options for care, ultimately helping them promote a more efficient professional practice [26,27,39-41]. Scientific studies are published; best practices are documented; and, however, they are slow to be implemented. We are trying to evaluate if an innovative solution would allow for the application and integration of this knowledge into clinical practice. Interventions supported by IT can promote the creation, access, and application of clinical recommendations, care protocols, and regulations, as well as best practices, bringing KT to practice.

IT–enabled CoP can support learning and knowledge sharing within health care teams as well as promote best practices in a variety of clinical areas [26,27,40-42]. Wikis are being used to encourage and make it easier for clinical practitioners to share their knowledge and expertise [43-45]. Wikis can also help users adapt their knowledge for local contexts, making it more relevant and user-friendly [46,47] and to encourage collaboration between patients and clinical practitioners when developing support tools for patients [48,49]. As such, several authors suggested exploring collaborative Web-based platforms to share, create, and update content of clinical decision-assistance systems [46,50-53].

Systematic reviews have demonstrated the value of support tools for integrated clinical approaches [54]. Studies have also shown that the mere availability of Web-based resources is not sufficient. Even if clinical practitioners could reliably find the answers to their questions in about 50% of cases, for some reason, they do not follow through on the inquiry. Researchers suggest that technological solutions should provide access to high-quality evidence, as per the just-in-time principles, in the clinical decision-making process [55].

### Technological Solution: Software for the Evolution of Knowledge in MEDicine

An innovative platform was created in the Outaouais Region in Quebec, Canada, in 2014. Initially used in the emergency area, this platform is now being assessed at the institutional level and is an integral part of the Centre intégré de santé et des services sociaux de l'Outaouais and Relations and Educational Research Department (RERD) approach. Software for the Evolution of Knowledge in MEDicine (SEKMED) is an interactive and dynamic working Web platform employing a multidimensional approach to knowledge, which considers the various dimensions linked to clinical practice such as scientific, organizational, professional, and experiential. The solution also allows collaboration and interactions through an iterative and continuous process of knowledge generation supported by the involvement of CoP.

The platform aims to facilitate the coordination of efforts deployed by members of a CoP, the accreditation and standards bodies, within an advanced Wiki-type tool dedicated to the creation, aggregation, and updating of interactive resources supporting the patient’s clinical history intake, physical examination, differential diagnosis consideration, take over, and therapeutic or orientation processes by the clinical practitioners. Moreover, these resources are, then, made available, as per the just-in-time principles, directly in the clinical practitioners’ processes using an ontological recognition engine which recognizes the terms associated with resources.

SEKMED assists clinical practitioners in their efforts to stay up-to-date and enables the integration of best practices as well as a better use of diagnostic and therapeutic resources. This project is destined to clinical practitioners but it can be interfaced with other health professions. There is an introductory video about the SEKMED platform [56].

**Figure 1:**
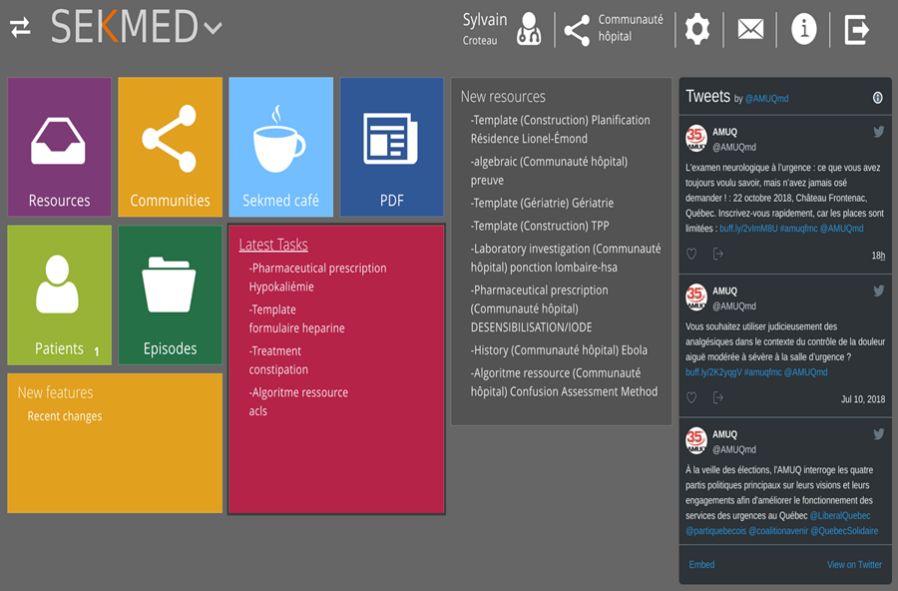
View of the platform dashboard.

Figure 1 illustrates the dashboard of SEKMED. The platform is designed in 9 sections.

Note that every section of the menu could be accessed by clicking in the SEKMED logo so that we can choose the appropriate section.

SEKMED aims at giving the clinician a representation of all the different elements that constitute its practice and facilitate the exchange with the community they are a part of. Those elements are represented in the different sections of the platform and are represented in the dashboard that we see in Figure 1. We will give a brief description for those sections, but the main focus of SEKMED is to facilitate the creation of interactive resources that support the different clinical processes, the discussion of those resources to improve them and validate them at a higher level, and to give just-in-time access to high-quality evidence in the context of patient care decision making.

The following is a brief description of every section:

1. Resources: Here we can search for specific resources in one community or in other communities. Resources are built by the community with tools that are provided. They are formatted in a way to be immediately clinically useful. A resource could be an interactive template for history taking, physical examination, investigation, treatment, and recommendation to patients. It can also be references or educational videos that are provided in the clinical process, following the just-in-time principle. Filters could be applied during that search (type of resource, owner of the resource, and community). This process is completely distinct from the discovery of resources in the clinical setting. It is also where one can decide to add a resource. It is where the process of creation happens.
2. Communities: A list of all communities, my own communities, and pending request to adhere to one. The concept of community is important when we understand that the content that will be made available to the clinician is dependent on the fact that he or she is a member of one of them specifically.
3. SEKMED Café: A section where you can interact with your community. (Chat, message from the community, from the center where the clinician is working, a forum for longer discussion around subjects proposed by the members, a humor section, a list of the members, and a means of communication between members).
4. PDF: A section facilitating storage of PDF. From there, they can be made available to the communities. Note that most resources are not PDF, but it is often the start of the evolution from a more static to fully interactive template.
5. Patients: Index of existing patients.
6. Episodes: When a patient is selected, an episode is created for every specific encounter. Here, we can find a list of the still opened episodes, or search for one based on different criteria.
7. Latest Tasks: A list of the resources a community would like to see implemented. Each task links to a real document. This document only has a title and, in some cases, instructions for specific objectives associated. This document, in task, is then attributed or available to the members of the community of practice as a specific project.
8. New resources: A list of the most recent resources created.
9. Tweets: The Twitter feed of the community.

**Figure 2:**
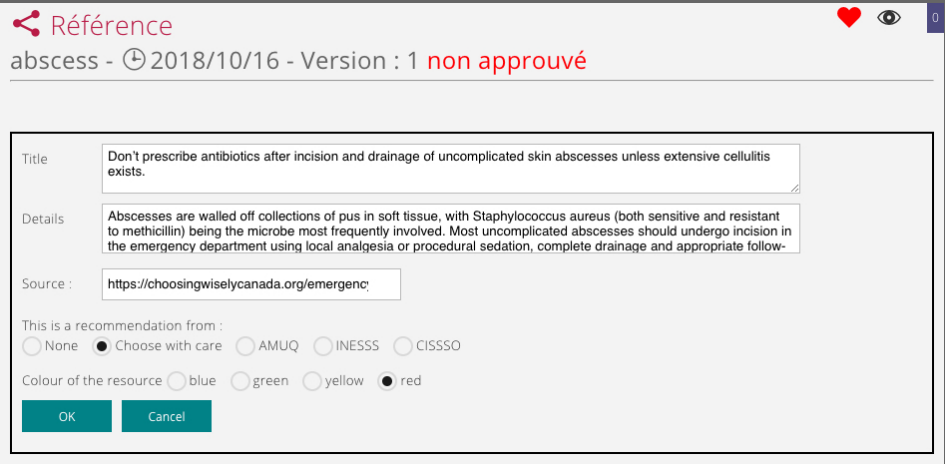
Integration of clinical recommendation.

The SEKMED platform allows the creation of different kinds of resources, permitting the integration recommendations from normative or accrediting bodies. As shown in Figure 2, we see the implementation of a compass kind of resources. This type of resources informs the clinician about one specific recommendation of the campaign, Choosing Wisely.

**Figure 3:**
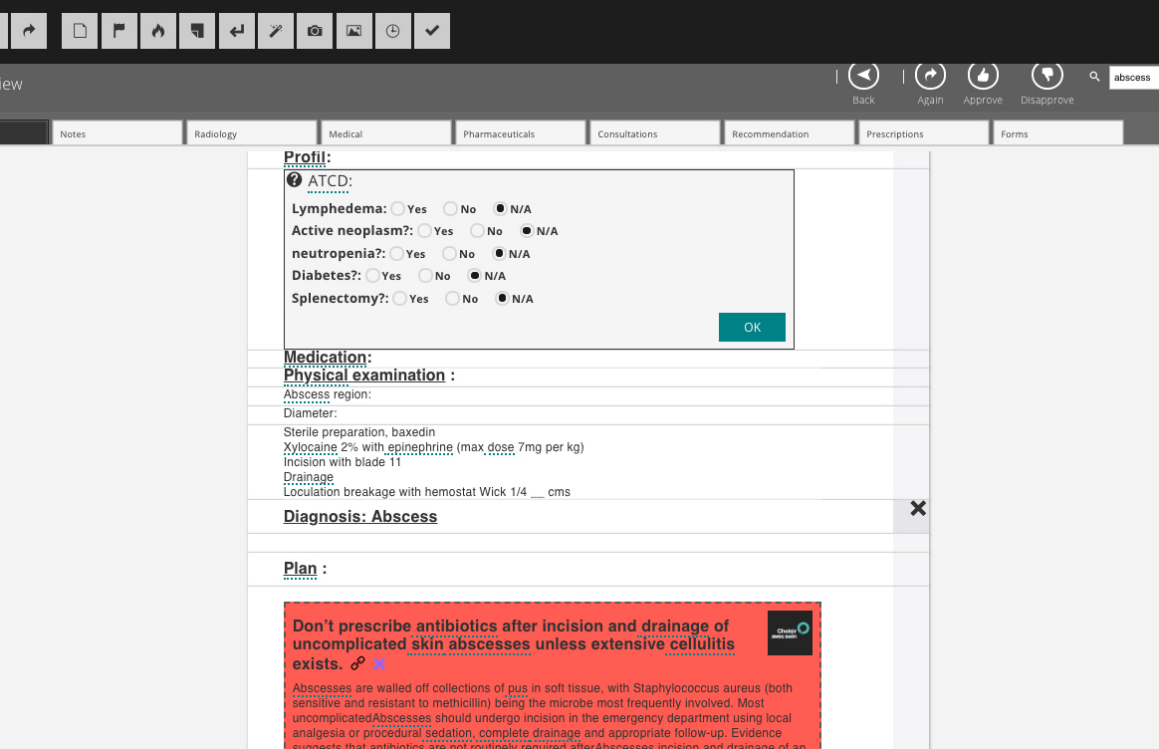
Template for abscess.

As shown in Figure 2, the title is the specific recommendation made by the normative body, the details represent the justification for the recommendation, there is and shall always be a link to the source of the information, a specific element of the resources permit the insertion of that link, there is a list of the normative or accrediting bodies from which to choose, this selection will also insert the logo of the later in the resource itself. There is a choice of color providing a visual clue as to the fact that something should not be done (red), should be done (green), or one should be careful about something (yellow), and there is also neutral powder blue for more information. That kind of resource is inserted into a template for a specific condition (Figure 3). When the template is used, the information reaches the clinician in its process. Figure 3 is a template for abscess with the usual questions, the technique for drainage, and the specific recommendation for not prescribing antibiotics in uncomplicated cases.

**Figure 4:**
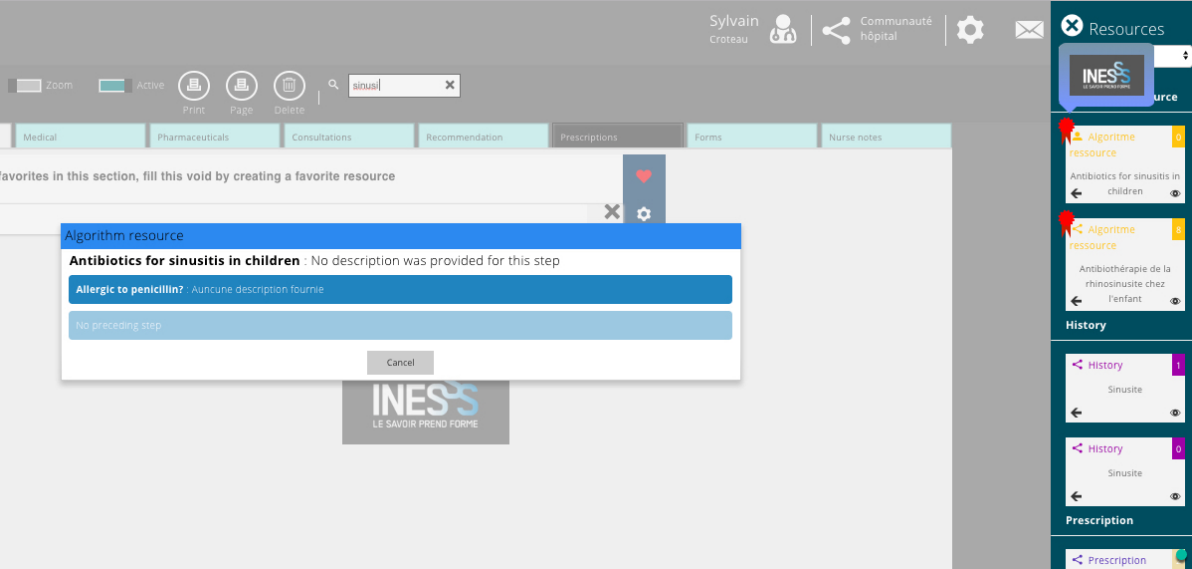
Recommendation from Institut national d’excellence en santé et en services sociaux (INESSS) for antibiotic treatment of sinusitis in children.

In certain cases, a full template or an algorithm could represent the recommendation of a normative body or an organization (Figure 4). It is then possible to identify resources that would have been validated at a high level by the application of a certificate. Here, a recommendation from the INESSS for the antibiotic treatment of sinusitis in children is presented as an algorithm. The certificate identifies those resources that come from normative of accrediting bodies in comparisons with the one that is created by the individuals in the community and have not gone through a process of intensive review and accreditation.

The certification needs to go to a review committee. The editing process is then blocked for the CoP. The certificate is set in advance, and the process for adding it is fairly simple.

### Objective

The main objective of the pilot project is to assess the usefulness of the SEKMED platform in the implementation of medical recommendations recognized by the members of CoP, accreditation bodies (Association des médecins d'urgence du Québec or AMUQ), a campaign such as Choosing Wisely and standards bodies (INESSS) in clinical practice in real health care settings within the province of Quebec, Canada.

As part of the project, SEKMED will be assessed with 3 CoP: (1) emergency physicians, (2) general medicine patient care unit physicians, as well as (3) external students and residents studying family medicine. Group 1 will be composed of 20 emergency physicians from the Hull Hospital and the Gatineau Hospital. Group 2 will have 20 patient care unit physicians from the Hull Hospital, and the third group will consist of approximately 10 external students and residents in family medicine from McGill University.

## Methods

### Validation and Evaluation Methodology in a Real Caregiving Situation

The project will focus strictly on (1) the research over a 24-month period, (2) the ethical and scientific endorsement of the project; (3) the employment of students; (4) the recruitment and training of participants; (5) the initial implementation of the 6 specific objectives through the analysis and interpretation of quantitative and qualitative data generated during the study; (6) continuous improvement; and (7) the dissemination of results within health care work environments as well as stakeholders and academic audiences.

**Figure 5:**
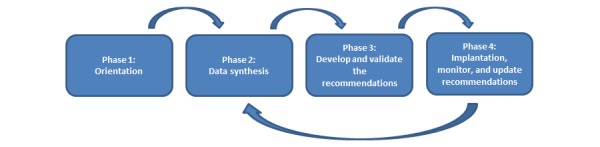
Research program.

The project will be completed in 4 phases (Figure 5). The knowledge gained during each phase will be applied on an iterative and continuous basis to all other phases over a 2-year period.

#### Phase 1: Orientation

This will consist in the consultation and mobilization of participants in the 3 CoP to better understand the culture, practical settings, values, and preferences, as well as to identify their local decision-making needs at the clinical and practical level. Three work teams will then be created to represent each CoP: (1) emergency physicians; (2) patient care unit physicians; as well as (3) external students and residents in family medicine. Each community will be represented by a voluntary champion. All participants will complete training during this phase to familiarize themselves and master the SEKMED platform’s functions. Each will receive a user guide with up-to-date content presenting among other things, video clips and screenshots to ensure a better understanding of the platform.

#### Phase 2: Data Synthesis

This will aim to rigorously collect, integrate, and synthesize the recommendations provided by the members of each community of practice, accreditation bodies (AMUQ), and standards bodies (INESSS and Choosing Wisely). Recommendations are based on 3 types of data: scientific, contextual, and experiential. An initial information search will be conducted with each team to identify the educational and informational content, templates, forms, follow-up sheet, decision-making algorithms, and other relevant resources used by the CoP. A second information search will be undertaken with accreditation and standards bodies only with the vision to compile a comprehensive list of recommendations for each specialty.

#### Phase 3: Develop and Validate the Recommendations

This will consist in mobilizing work teams to (1) collect and integrate scientific information, within the implementation context (contextual data) and practical experience context (experiential data), and better understand the values and preferences of each community of practice; (2) verify if the recommendations are available or not. If they are available via the accreditation and/or standards bodies, they can be considered as they stand or be adapted for local usage; (3) propose recommendations in a concise manner that are lacking and have not been listed by members of each CoP, by accreditation bodies, or by standards bodies but are deemed useful, relevant, acceptable, and applicable in the field by teams; (4) select recommendations from a list provided by accreditation and standards bodies that meet the CoP’s clinical and practical needs; and (5) review and validate all recommendations that are considered essential to the practice and relevant to be applied and monitored on the SEKMED platform (Phase 4).

#### Phase 4: Implantation, Monitor, and Update Recommendations

In the final phase of the project, recommendations will be implemented, monitored, and updated. The implementation step aims to integrate, disseminate, and transfer recommendations across the SEKMED platform so that users may use them in their own CoP. Implementation support strategies will be available at this stage to promote ownership and facilitate the dissemination of the recommendations. The monitoring step will measure the degree of implementation of recommendations and their impact on clinical practices, care, and services management as well as the health and well-being of populations. Finally, the last step consists in making regular updates with working teams, ensuring continuous access and use of the best evidence and the recommendations provided by accreditation and standards bodies in clinical practice by sharing and making them available. Following phase 4, there will be an iterative and continuous process, starting at phase 2, throughout the project.

### Data Collection Methods

The 4 phases described above will enable us to meet our 6 specific research objectives. The recommended data collection methodology aims to achieve deliverables based on qualitative and quantitative methods. This triangulation of methods is vital for an understanding of complex phenomena, and it allows data enhancements, questioning, monitoring, and verification [57]. Some authors suggest that the triangulation of data sources, which compares data produced by 2 or several different and independent methods, increases the interpretive power [58]. This method, whether parallel or sequential, seeks using different measures and observations, to reduce bias in each method. The goal is to exploit the complementary nature of methodological processes to get the very best out of them.

#### Specific Objective 1

This objective was to measure the integration of recommendations in clinical practices. This objective will demonstrate through SEKMED the uptake of medical recommendations in clinical practices. This will enable us to determine if users really take ownership and integrate the recommendations into their daily work. To do so, we will export the SEKMED data in a Microsoft Excel file to produce descriptive statistics. The information provided by the data will allow us to measure the monthly progress of the following indicators:

1. The number of recommendations used by each physician in clinical practice.
2. Percentage of specific recommendations used at specific clinical situations,
3. Percentage of physicians who are still using recommendations in clinical practice.

#### Specific Objective 2

This objective was to measure the before and after improvements in practices. The objective aims to compare improvements in practices, before and after the intervention, by using medical recommendations. We will be using the same approach as Specific Objective 1 for the data extraction, analysis, and processing.

The following indicators will be measured:

1. The number of new recommendations integrated into the platform that is used by physicians;
2. Average and median time required for the integration of recommendations in a work environment.

#### Specific Objective 3

This objective was to measure the value attributed by physicians to recommendations. This objective will appraise the overall value (experience and satisfaction) attributed by physicians to recommendations provided by members of the community of practice, as well as accreditation and standards bodies in their field of practice. A total of 4 focus groups will be held during the project. The data collected with the first 2 specific objectives will be discussed at each focus group. This approach aims to foster reflective practices in providing an opportunity to step back and collectively review the experiences and the participants’ point of view on this practice and how it facilitates the KT process.

Our approach must be flexible to produce the desired change and to adapt to the situation while allowing knowledge development, experience sharing, and ideas exchange to ultimately find solutions to common health care problems [59-61]. The assessment of this intervention will be completed using a mixed approach, which combines a qualitative approach with a quasi-experimental–type quantitative approach focused on the measured experience and satisfaction levels. Using this approach, we will be able to determine the extent to which reflective learning can facilitate the transfer and use of recommendations made by the CoP.

#### Specific Objective 4

This objective was to measure the user’s platform experience. This objective will measure the user’s experience with the platform. The user experience is defined as an individual’s perception and response resulting from his or her use or the anticipated use of a system [62]. This understanding of the user experience is also essential in nature for these organizations who wish to offer interfaces that meet the various users’ evolving needs in an efficient manner [63]. As part of our project, we are developing a conceptual model of the user experience based on the rich conceptual framework of IT usage prepared by Barki, Titah, and Boffo [62], which takes simultaneously into account the characteristics of the technology (eg, usability), the user (eg, expertise), and the task to achieve (eg, complexity) to better understand the concept of utilization. We will also integrate technology acceptance and utilization models based on behavioral intents which are influenced by IT usage perceptions and its usability [63-66].

The research team will administer a questionnaire after each experimental period with the tool. We have chosen to analyze the data using the partial least squares structural equation modeling (PLS-SEM) to verify and refine the proposed theoretical models. PLS-SEM is a second generation multivariate statistical analysis method. Although first generation techniques usually rely on traditional research statistical methods such as regression and analysis of the variance, second generation techniques compensate the first generation techniques’ shortcomings by notably taking the errors in measurement into account. PLS is relevant for our project because it is used for exploratory assessment purposes such as the analysis of trends and the identification of relationships.

#### Specific Objective 5

This objective was to measure the educational benefits according to medical and external students. This objective consists of assessing the attitude and intent of external students and residents in family medicine toward the educational benefits of SEKMED. We will administer the same questionnaire used for Specific Objective 4. We will also organize focus groups to learn about the user’s experience in the adaptation and learning process. A total of 4 focus groups will be held during the project.

Several studies have shown that IT promotes the adoption of a pedagogical approach which places the student or the learner at the center of the learning process. IT indeed provides the innovative means, not only for the dissemination of knowledge, but also for the exploration of learning strategies promoting competency development—access to information, real-time communication, and exchange with CoP. Many papers focus on experimentation with a Wiki, but fewer studies explain the rationale and the pedagogical foundations [67,68]. Several authors think it is possible to improve the teaching system on clinical reasoning by using techniques that are more efficient than conventional teaching [69]. We wish to demonstrate that SEKMED enables learning, including accompanying and complementary skills evaluation support by governing the problem-solving process and by assessing the students’ and residents’ ability to process the information. In that respect, SEKMED could be used as a diagnostic assessment in providing a more personalized support program to learning.

#### Specific Objective 6

This objective was to measure the organizational benefits according to stakeholders. This objective will assess the attitude and intent of the RERD and the stakeholders toward the changes resulting from the use of the platform and the achievement of their specific objectives. In view of the specificity of this objective and the target audience, an interview guide will be developed and validated before its use. The interview guide will measure the attitude and intent of the RERD and the stakeholders toward the changes resulting from the use of SEKMED and the achievement of their specific organizational objectives.

RERD will benefit from a tool facilitating the transfer, mobilization, and validation of knowledge gained within the organization while driving innovation. In support of all management bodies governing the clinical practice, they will be able to monitor medical and social interventions more rigorously in real time. Statistical functions and the ability to extract granular data actually make it possible to assess all medical acts, track changes, and implement best practices while also validating the application and the relevancy of the recommendations issued by the organization or the accreditation and standards bodies. In other words, all actions performed by caregivers become traceable and measurable.

Stakeholders supporting the clinical practices are the Directorate of Professional Services, Directorate of Multidisciplinary Services, and the Nursing Directorate. As mentioned above, SEKMED has the ability to extract data at the granular level, which reduces the amount of time they require to complete specific tasks. As an example, the review of medical records, also known as audits, will be improved owing to the ability to track all actions performed by a health care professional. SEKMED also promotes the dissemination, among clinical practitioners, of clinical tools and best practices, thus simplifying their implementation and training needs. To conclude, these data allow the development of follow-up and performance indicators that will facilitate the monitoring activities performed by management bodies supporting the clinical practice.

## Results

This project was funded in April 2018 by the Fonds de soutien à l’innovation en santé et en services sociaux for 24 months. Ethics approval has been attained, the study began in June 2018, the data collection will be complete at the end of December 2019, and the data analysis will start in winter 2020. Both major city hospitals in the Outaouais region, Quebec, Canada, have agreed to participate in the project.

## Discussion

If results show preliminary efficacy and usability of the system, a large-scale implementation will be conducted.

The expected benefits generated by this protocol on the improvement of care and service delivery are as follows:

1. The use of better evidence and recommendations issued by the accreditation and standards bodies in clinical practice, by continuously sharing and making them available within the context of their implementation.
2. To gain efficiencies in the dissemination, usage, and update of institutional protocols.
3. Gaining efficiencies for clinical practitioners.
4. The rationalization of the resource and budget use.
5. The efficient harmonization of practices.
6. The improvement of the teaching quality.
7. The use of granular data to assess the quality of the act as well as administrative and research purposes.

## Appendix

Multimedia Appendix 1 Peer-review report from the granting agency.

Multimedia Appendix 2 Scientific validation from the Scientific Committee of the Centre intégré de santé et des services sociaux de l'Outaouais in support of the peer-review report from the granting agency.

## Abbreviations

AMUQ: Association des médecins d'urgence du Québec
CIHR: Canadian Institutes of Health Research
CoP: Communities of practice
INESSS: Institut national d’excellence en santé et en services sociaux
IT: information technology
KT: knowledge translation
PLS-SEM: partial least squares structural equation modeling
RERD: Relations and Educational Research Department
SEKMED: Software for the Evolution of Knowledge in MEDicine
