# Supplementary material for: An Evidence-Based Health Care Knowledge Integration System: Assessment Protocol
Source: JMIR Res Protoc. 2019 Mar 11;8(3):e11754. doi: 10.2196/11754 (PMC6431825; doi:10.2196/11754)
Supplement: Multimedia Appendix 1 [file resprot_v8i3e11754_app1.pdf]

MEDTEQ
L'INNOVATION POUR LA SANTÉ
INNOVATION FOR HEALTH

Partenaire financier :

Économie, Science et Innovation Québec

Montréal, le **15 février 2018**

Objet : Fonds de soutien à l'innovation en santé et services sociaux – projet **1-01** intitulé ***« SEKMED : plateforme d'aide à la démarche clinique supportée par les communautés de pratique »*** de **Solution Doc 2.0** avec le **CISSS de l'Outaouais.**

Mesdames/Messieurs,

Merci d'avoir participé au premier Appel à propositions du Fonds de soutien à l'innovation en santé et services sociaux – FSISSS – lancé le 9 novembre 2017. Nous sommes ravis de vous informer que le Ministère de l'Économie, de la Science et de l'Innovation (MÉSI) a approuvé en principe l'octroi d'une subvention au CISSS de l'Outaouais, pour la demande susmentionnée.

L'octroi de la subvention est conditionnel à la signature d'une entente de financement entre Solution Doc 2.0 et le ministère avant le 1er mars 2018.
Le versement des fonds à Solution Doc 2.0 sera conditionnel à :

- l'obtention des approbations éthiques nécessaires auprès des instances désignées, le cas échéant ;
- l'adhésion en règle de Solution Doc 2.0 au Consortium MEDTEQ pour toute la durée du projet ;
- le versement des frais annuels d'administration du CISSS de l'Outaouais à MEDTEQ;
- la signature d'une seconde entente entre Solution Doc 2.0 et le CISSS de l'Outaouais avant le 1er juin 2018.

À titre d'information, voici les commentaires reçus suite à l'évaluation de votre demande :

Admissibilité :

Les contributions en nature d'établissements de santé ne font pas partie du montage financier. Avec le montage actuel, la demande de financement est à hauteur de 58%, et non de 50%. Les contributions devront être corrigées en conséquence dans l'entente de financement.

Forces :

-La pertinence et la qualité des partenaires et de l'équipe du projet.
-Impacts positifs potentiels pour la PME, pour les pratiques médicales et pour les soins aux patients.
-Besoin réel et important.

-Contexte du projet et problématique très bien documentés (aider les médecins à mettre en place les pratiques exemplaires).
-Aide à la démarche clinique.
-Développer en étroit partenariat avec les médecins -implication de leaders cliniciens de 2 sites : urgences et unité de soins.
-Facilitera l'appropriation et utilisation de cette technologie par les 60 médecins visés.
-Implication de plusieurs partenaires clés à l'échelle de la province, dont AMUQ et le CMFQ.
-PI a l'expertise pour mener ce projet en étroit partenariat avec une équipe diversifiée, dont Véronique Nabelsi

Faiblesses :

-La principale faiblesse est de décrire l'outil d'aide à la décision, son implantation dans le milieu ciblé, et ensuite, de présenter les objectifs du projet de recherche en cohérence avec ce développement technologique. La pertinence de l'outil est présentée de manière très habile. Ensuite, on a peu de détails du devis évaluatif.
-Les objectifs spécifiques sont peu développés. Quelles connaissances visent ces objectifs? Cette section mériterait à être bonifiée.
-Les modalités de fonctionnement et le caractère innovant de la plateforme logicielle ne sont pas clairs.
-Les interventions et les métriques qui seront évaluées ne sont pas claires.
-Besoin d'établir les statistiques requises pour quantifier l'efficacité du milieu de soins réel de la plateforme logicielle.
-Comment cela s'intègre-t-il avec le dossier patient local et le DSQ ?
-Comment est fait le lien avec des initiatives telles que « choosing wisely » ? L'initiative est mentionnée, mais pas son intégration.
-Pas de réelle évaluation des bénéfices économiques pour le réseau de la santé.
-Le plan marketing n'est pas clair.
-Embauche d'étudiants au doctorat 21h/semaine. Est-ce que ce projet s'imbrique dans leurs études ou ce projet est complémentaire? Si ce projet est complémentaire, 21h/semaine à ce projet risque de compromettre la durée de leurs études.
-Note : la HAS n'est pas européenne, mais française.

Prochaine étape :

Afin de commencer le processus de conclusion de l'entente de financement, **merci d'entrer rapidement en contact avec Madame Marie Bouillé**, conseillère au MÉSI, à l'adresse suivante : Marie.Bouille@economie.gouv.qc.ca.

Veuillez agréer, Mesdames/Messieurs, l'expression de nos sentiments les meilleurs.

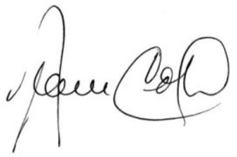

Diane Côté
Présidente-directrice générale de MEDTEQ
