# Supplementary material for: An Evidence-Based Health Care Knowledge Integration System: Assessment Protocol
Source: JMIR Res Protoc. 2019 Mar 11;8(3):e11754. doi: 10.2196/11754 (PMC6431825; doi:10.2196/11754)
Supplement: Multimedia Appendix 2 [file resprot_v8i3e11754_app2.pdf]

Centre intégré
de santé
et de services sociaux
de l'Outaouais
Québec

Direction de l'enseignement, des relations universitaires et de la recherche

Validation scientifique d'un projet de recherche

Version 2017-07-12

Date d'évaluation : 2018-02-26

Chercheur principal ou local : Véronique Nabelsi

Établissement d'affiliation : UQO

Titre du projet : SEKMED : plateforme d'aide à la démarche clinique supportée par les communautés de pratique

A – Évaluation scientifique externe (documents requis à l'appui)	
Le projet a obtenu une validation scientifique dans un établissement de santé par un conseil scientifique ou un comité d'éthique qui cumule la fonction d'évaluation scientifique.	☐
Le chercheur a obtenu une subvention d'un des trois conseils (CRSH, IRSC, CRSNG) ou des FRQ pour mener sa recherche.	☐
Le chercheur étudiant soumet un projet de thèse soutenu et approuvé devant un comité de thèse.	☐
Le chercheur étudiant soumet un projet de maîtrise (sans comité d'évaluation).	☐
Le chercheur a reçu une subvention à la suite d'un concours.	☒
Si l'une des situations ci-dessus s'applique, ne pas remplir la section B et passer à la section « Décision du Conseil scientifique »	

B – Évaluation scientifique du projet de recherche par le Conseil scientifique	Oui	Passable	À réviser	Sans objet
1. Une section « Introduction » et/ou « Cadre théorique » et/ou « Recension des écrits » et/ou « Mise en contexte » soutient bien l'argumentaire, la démarche, les visées de la recherche ou les résultats attendus.	☐	☐	☐	☐
2. Les objectifs sont clairement identifiés.	☐	☐	☐	☐
3. L'hypothèse/la question de recherche est clairement formulée.	☐	☐	☐	☐
4. Les candidats recherchés sont clairement décrits (caractéristiques, critères d'inclusion et/ou d'exclusion) et le nombre (adapté à la méthodologie) est justifié.	☐	☐	☐	☐
5. Le devis de recherche est approprié pour répondre aux objectifs ou hypothèses ou questions de recherche.	☐	☐	☐	☐
6. La procédure est adéquatement décrite.	☐	☐	☐	☐
7. La méthodologie utilisée (qualitative, quantitative ou mixte) pour la collecte des données est clairement décrite et justifiée.	☐	☐	☐	☐
8. La qualité psychométrique des instruments est discutée (ou il est fait mention qu'ils sont en cours de validation) lorsqu'applicable	☐	☐	☐	☐
9. L'analyse des données (qualitatives, quantitatives ou mixtes) est spécifiée et appropriée.	☐	☐	☐	☐
10. L'étude est susceptible de produire des résultats qui pourraient être publiés.	☐	☐	☐	☐

500, boulevard de l'Hôpital, bureau 202
Gatineau (Québec) J8V 2P5
Téléphone : 819 966-6171, poste 3823
Télécopieur : 819 966-6389
www.cisss-outaouais.gouv.qc.ca

DÉCISION DU CONSEIL SCIENTIFIQUE

A – Évaluation scientifique externe

☒ Évaluation scientifique externe reconnue par le Conseil scientifique.

COMMENTAIRES

La présidence du Conseil scientifique a reçu, analysé et approuvé les documents requis. Il est recommandé que la chercheure réponde aux commentaires formulés par MEDTEC en vue de clarifier les objectifs, les procédures et l'évaluation du projet.

B – Évaluation scientifique du projet de recherche par le Conseil scientifique

☐ Approuvé

- ☐ Lors de l'évaluation, les commentaires ci-dessous ont été formulés, mais ont somme toute mené à une décision favorable. Vous pouvez en tenir compte pour bonifier votre projet.

☐ Approuvé conditionnellement aux modifications mineures demandées (voir encadré plus bas).

☐ Refusé. Nécessite des modifications majeures (voir encadré plus bas).

COMMENTAIRES et/ou DEMANDES DE MODIFICATIONS

Bon succès dans votre projet!

Marguerite Soulière, Ph. D.
Présidente du Conseil scientifique
Centre de recherche du CISSS de l'Outaouais

500, boulevard de l'Hôpital, bureau 202
Gatineau (Québec) J8V 2P5
Téléphone : 819 966-6171, poste 3823
Télécopieur : 819 966-6389
www.cisss-outaouais.gouv.qc.ca

De : Nabelsi, Véronique <veronique.nabelsi@uqo.ca>

Envoyé : 26 février 2018 17:30

À : Manon Dupuis (CISSSO)

Objet : RE: Projet 114

Pour info, l'organisme subventionnaire, MEDTEQ, n'avait pas lu mon annexe 2 sur les objectifs spécifiques avec les mesures et les précision.

Je n'ai pas besoin de faire de modification à cet égard.

Véronique
